# Time–Frequency Signatures of Electronic Coherence of Colloidal CdSe Quantum Dot Dimer Assemblies Probed at Room Temperature by Two-Dimensional Electronic Spectroscopy

**DOI:** 10.3390/nano13142096

**Published:** 2023-07-18

**Authors:** James R. Hamilton, Edoardo Amarotti, Carlo N. Dibenedetto, Marinella Striccoli, Raphael D. Levine, Elisabetta Collini, Francoise Remacle

**Affiliations:** 1 Department of Theoretical Physical Chemistry, University of Liège, B4000 Liège, Belgium; james.hamilton@uliege.be; 2 Department of Chemical Sciences, University of Padova, 35131 Padova, Italy; edoardo.amarotti@chemphys.lu.se (E.A.); elisabetta.collini@unipd.it (E.C.); 3 CNR-IPCF SS Bari, c/o Chemistry Department, University of Bari Aldo Moro, 70126 Bari, Italy; c.dibenedetto@ba.ipcf.cnr.it (C.N.D.); m.striccoli@ba.ipcf.cnr.it (M.S.); 4 Chemistry Department, University of Bari Aldo Moro, 70126 Bari, Italy; 5 The Fritz Haber Research Center for Molecular Dynamics, Institute of Chemistry, The Hebrew University of Jerusalem, Jerusalem 91904, Israel; raphy@mail.huji.ac.il

**Keywords:** 2D femtosecond electronic spectroscopy, photocurrent action spectroscopy, CdSe quantum dot dimers, electronic coherences in quantum dot dimers, quantum technologies

## Abstract

Electronic coherence signatures can be directly identified in the time–frequency maps measured in two-dimensional electronic spectroscopy (2DES). Here, we demonstrate the theory and discuss the advantages of this approach via the detailed application to the fast-femtosecond beatings of a wide variety of electronic coherences in ensemble dimers of quantum dots (QDs), assembled from QDs of 3 nm in diameter, with 8% size dispersion in diameter. The observed and computed results can be consistently characterized directly in the time–frequency domain by probing the polarization in the 2DES setup. The experimental and computed time–frequency maps are found in very good agreement, and several electronic coherences are characterized at room temperature in solution, before the extensive dephasing due to the size dispersion begins. As compared to the frequency–frequency maps that are commonly used in 2DES, the time–frequency maps allow exploiting electronic coherences without additional post-processing and with fewer 2DES measurements. Towards quantum technology applications, we also report on the modeling of the time–frequency photocurrent response of these electronic coherences, which paves the way to integrating QD devices with classical architectures, thereby enhancing the quantum advantage of such technologies for parallel information processing at room temperature.

## 1. Introduction

Semi-conducting nanoparticles, or quantum dots (QDs), are a promising hardware for a wide variety of quantum technologies [1,2,3,4,5,6,7]. Recently, we proposed to exploit the femtosecond fast-beating electronic coherences in small, few-nm QDs and QD dimers for implementing quantum parallel information processing at room temperature [8,9,10,11], using electronic coherences as logic variables. Our scheme offers a significant quantum advantage, as for a set of *N* coupled quantum states, *N^2^* − 1 coherences can be used to process information in parallel [8,12].

**Figure 1:**
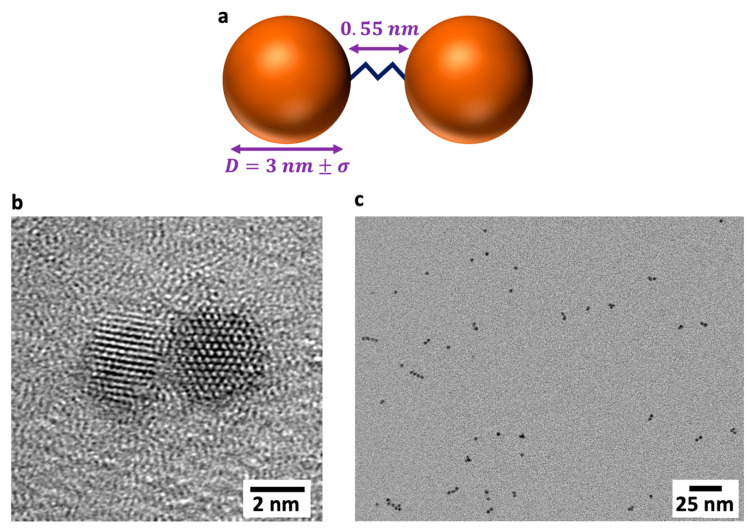
(**a**) Schematic representation of a CdSe quasi-homodimer. The dimer is assembled by covalently bonding two QDs with a 0.55 nm $S{\left({CH}_{3}\right)}_{2}S$ ligand. The QDs are drawn from an ensemble with a mean diameter, $\bar{D}$, of 3 nm and size dispersion, σ. (**b**) A high-resolution transmission electronic microscopy (HR-TEM) image of one of the quasi-homodimers prepared for this work. (**c**) A larger TEM micrograph of the CdSe QDs dimers.

**Figure 2:**
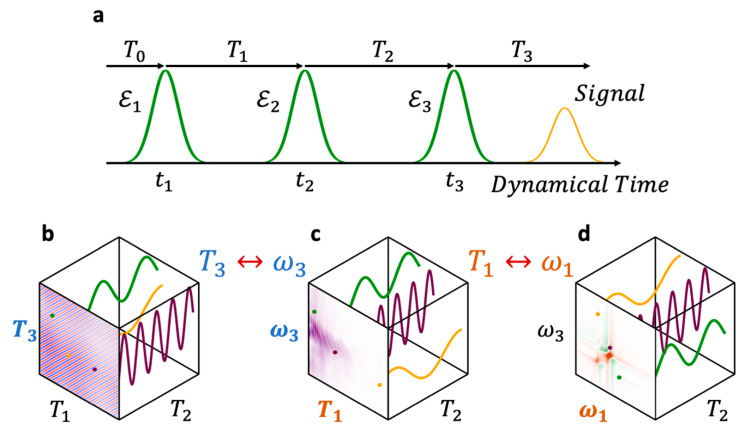
(**a**) Time ordering of the three fs laser pulses as used in the 2DES experiment and in the modeling. (**b**–**d**) Different possible representations of the final three-dimensional signal obtained after a 2DES measurement (or of modeled data). The final cube of polarization response data can be cast as a function of: (**b**) the three time delays between pulses (*T*_1_, *T*_2_, *T*_3_) (Equation (4)), and (**c**) the first two time delays between pulses and the third delay in the frequency domain (*T*_1_, *T*_2_, $\omega_{3}$) (Equation (8)). These are the data studied in this work. (**d**) The first and third delays in the frequency domain and the second delay in the time domain ($\omega_{1}$, *T*_2_,$\;\omega_{3}$). This is the representation typically used when 2DES measurements are published, as was the case in [7]. In panels (**b**–**d**), the green, purple and orange lines schematically represent the beatings of three different electronic coherences along *T*_2_.

In previous joint theoretical–experimental studies [7,13,14,15], we reported how electronic coherences could be observed and tuned in ensembles of small (mean diameter, $\bar{D}$ = 2.5 to 3 nm), size-dispersed (σ = 5% to 9%) CdSe QDs and QD quasi-homodimers (Figure 1), addressed in a two-dimensional electronic spectroscopy (2DES) BOXCARS setup (see Supplementary Materials Section S1). In this setup, the polarization response is measured as a function of the first two delay times, *T*_1_ and *T*_2_, and directly in the frequency domain, $\omega_{3}$, of the third delay time, *T*_3_, using a CCD camera (see Figure 2a). A Fourier transform along the first delay time, *T*_1_, brings the maps into the frequency domain, $\omega_{1}$, and allows retrieving the conventional 2DES response as a function of ($\omega_{1}$, $T_{2},\;\omega_{3}$), as shown in Figure 2d [5,16,17,18]. A good agreement was found between the computed and experimental coherence responses of small ≈3 nm QDs and QD dimers along *T*_2_ traces of points extracted at different coordinates in the frequency ($\omega_{1}$, $\omega_{3}$) maps [13,14,15]. 

The ($\omega_{1},\omega_{3})$ frequency maps of the ($\omega_{1}$, $T_{2},\omega_{3}$) response provide ($\omega_{1}^{i},\omega_{3}^{j})$ coordinates for coherences between pairs of excited electronic states (*i,j*) evolving along *T*_2_, where $\omega_{1}^{i}$ corresponds to the excitation of state *i*, $\omega_{1}^{i}=\omega_{i}-\omega_{GS}$, and $\omega_{3}^{j}$ is the emission from state *j*, $\omega_{3}^{j}=\omega_{j}-\omega_{GS}$. Bringing *T*_1_ to the frequency domain, however, requires a large sampling of this delay time, as well as computationally expensive FFT post-processing [19]. Here, we show, by comparing experimental and computed data for the BOXCARS setup [20], that the electronic coherences can be equally accurately probed along *T*_2_ traces in time–frequency (*T*_1_, $\omega_{3}$) polarization maps, which require a smaller number of 2DES measurements and less post-processing. In ($T_{1}$,$\;\omega_{3}$) time–frequency maps, there can be several coherences between excited states that beat along *T*_2_ at a specific address ($T_{1}$, $\omega_{3}$) in the map. The reason is that the coherences along *T*_2_ are only partially resolved: they are resolved along $\omega_{3}$ but not along *T*_1_. All the coherences between the excited states *i* and *j,* that have a common state *j* that emits at the frequency $\omega_{3}^{j}$ and a different state *i,* will beat at the same address ($T_{1}$, $\omega_{3}$) in the map. Therefore, by measuring a single point in the ($T_{1}$, $\omega_{3}$) time–frequency maps, one can characterize a family of coherences that beat along *T*_2_. The ability to simultaneously process all these coherences enhances their potential for exploitation in parallel quantum information processing. 

The ability to measure electronic coherences in the directly measured data, without the need to compute full frequency maps, greatly enhances the potential for the use of electronic coherences of QDs in room-temperature quantum technologies. Towards applications to quantum information processing, the recently proposed photocurrent action-based setup [21,22,23,24,25,26,27,28] presents several advantages over the BOXCARS one for 2DES: it is a collinear setup, easier to operate, and the output is a photocurrent that can be processed easier and integrated with a classical computer. It is, therefore, of interest to investigate how electronic coherences are probed in the photocurrent response. We show, computationally, that the electronic coherences can also be robustly probed with the action-based photocurrent response. 

## 2. Materials and Methods

### 2.1. Synthesis of 3 nm CdSe QDs and Assembly into Quasi-Homodimers in Solution

The colloidal CdSe QDs were prepared in solution by mixing cadmium and selenium precursors using a hot-injection technique [29] in the presence of long-alkyl-chain-coordinating agents. Such molecules act as an organic capping layer on the QD surface, helping the controlled and quasi-epitaxial growth of the nanoparticles, allowing their dispersion in organic solvents, and preventing their aggregation. This approach allows controlling the growth parameters, the precursor ratio, and the temperature of the synthesis, for preparing small, 2.5 to 3.5 nm in diameter, QDs with narrow size distributions varying from 5 to 9% in mean diameter, making any further purification procedure unnecessary [30]. More detailed information can be found in the Supplementary Materials Section S2 and in [31]. 

Quasi-homodimers of CdSe QDs, with mean diameter $\bar{D}$ = 3 nm and size distribution σ = 8%, are assembled in solution by bonding pairs of size-dispersed QDs with a short (≈0.5 nm) propanedithiol ligand (see [31] for details of this procedure and [7] for the details of the synthesis of the QD dimers used in this work). Due to the unavoidable size dispersion, the QD dimers are not identical nano-objects. A schematic representation of a QD quasi-homodimer constructed in this fashion is shown in Figure 1a, and a transmission electronic microscopy (TEM) image of one of the dimers prepared for this work is shown in Figure 1b. A larger TEM micrograph of the dimers is shown in Figure 1c. Not all the QDs in the ensemble will be bonded together with the ligand to form dimers, and therefore, the dimer solution will retain a proportion of single QD monomers. The ratio of dimers to monomers in the sample is estimated at approximately 60:40.

### 2.2. 2DES Experimental Methods

The 2DES experiment is implemented using a fully non-collinear setup, in which three fs laser pulses in the visible range are incident upon the dimer solution from different spatial directions, fulfilling the BOXCARS phase-matching conditions [19]. For the 2DES measurements of a QD dimer ensemble assembled into a solid-state multilayer device, see [7]. Additional details about the experimental conditions are reported in the Supplementary Material (SM) Section S1. The experiment probes the optical polarization of the ensemble as a function of the delay times between the pulses, or of their corresponding frequencies. By giving independent control over the delay times, the BOXCARS setup (see Figure S1 of the Supplementary Materials) allows the signal in the different phase-matching directions (PMDs) to be easily extracted. For a description of the experimental setup, see [19], and for details on this implementation, see [7], as well as the Supplementary Materials Section S1. 

The three exciting pulses and the final signal observation are separated in time by the delay times, as defined in Figure 2a. The delay time, *T*_0_, is the time interval between the arbitrary origin of the time axis, set as the center time of the Local Oscillator (LO), and the first pulse, centered at *t*_1_. The LO is the fourth pulse, used as a time reference and employed for heterodyne detection [19].

The first delay time, the excitation or coherence time (*T*_1_), is the time separation between the first and second pulses. The second delay time, the population time (*T*_2_), is the time between the second and third pulses, and the third delay time, the rephasing or emission time (*T*_3_), is the time between the third pulse and the observation. The time at which the measurement is performed is defined as:

(1) $$t\equiv T_{0}+T_{1}+T_{2}+T_{3}$$

The polarization response from the ensemble can be measured in specific PMDs as a function of the delay times ($T_{1}$, $T_{2}$, $T_{3}$). Repeated measurements with different delays produce a “cube” of data, in which the polarization in a particular PMD is stored as a function of the delay parameters. Figure 2b shows the polarization in the rephasing direction in the time domain, as a function of ($T_{1}$, $T_{2}$, $T_{3}$). Each of the delay times ($T_{1}$, $T_{2}$, $T_{3}$) can be brought to the frequency domain ($\omega_{1}$, $\omega_{2}$, $\omega_{3}$) by Fourier transform, leading to what is sometimes referred to as 3D electron spectroscopy [32]. Figure 2c shows the same response as Figure 2b, only in the time–frequency domain ($T_{1}$, $T_{2}$, $\omega_{3}$). This is the typical form of the data obtained as raw output of a BOXCARS experimental setup, such as the one used for this work. Indeed, as explained above, in a typical BOXCARS experiment, the signal is measured while scanning the time intervals *T*_1_ and *T*_2_, whereas the dependence on the third time interval is measured directly in the frequency domain, $\omega_{3},$ by the detector [16]. For ease of interpretation, the signal is then typically Fourier-transformed along *T*_1_ [5,16,17,18]. This leads to a representation of the same data as a function of the excitation and emission frequencies and the population time ($\omega_{1}$, $T_{2}$, $\omega_{3}$), as shown in Figure 2d. Regardless of the chosen representation, the electronic coherences between the excited electronic states of the QD dimers can be probed by analyzing the data in these cubes as a function of the population time, $T_{2}$, as illustrated in Figure 2b–d. Coherences in the frequency maps ($\omega_{1}$, $\omega_{3}$) (Figure 2d) were studied in our previous work [7], while here, we analyze the ($T_{1}$, $\omega_{3}$) time–frequency maps.

### 2.3. Theoretical Methods

We model the electronic structure of each QD from two-hole, one-electron single-particle states. These single-particle electronic states are calculated using an effective mass-**k.p** Hamiltonian [33,34,35,36], constructed for CdSe using the size distribution of the ensemble (see [13,14,15]). This approximation defines two-holes, one-electron mono-excitons, 1S and 2S, per QD. When the laser intensity is weak enough, the formation of bi-excitons, two-electron, two-hole states, can be neglected [15,37].

**Figure 3:**
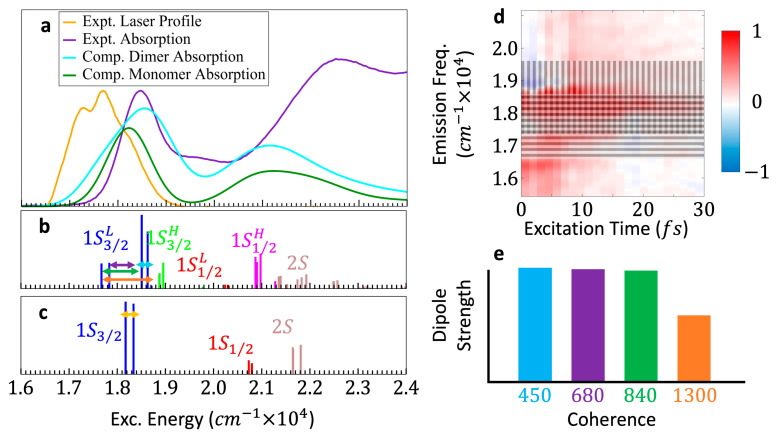
(**a**) Measured (violet) absorption spectra of the QD dimer sample and the calculated absorption spectra of the QD dimers (azure) and monomers (green), with the spectral profile of the laser pulse (orange) used for the 2DES measurement. (**b**) Calculated stick spectrum for the averaged dimer computed from an ensemble of 4000 3 nm/8% dimers. (**c**) Calculated stick spectrum for the averaged monomer computed from an ensemble of 4000 3 nm/8% monomers. The five electronic coherences between specific electronic FS states discussed in this work (four in the dimer, one in the monomer) are identified with horizontal, double-arrowed lines between their constituent FS states. (**d**) Inhomogeneous broadening of the four dimer electronic coherences in frequency, *ω*_3_. Patterns are superimposed onto a (*T*_1_, *ω*_3_) map, indicating the range of *ω*_3_ in which the four dimer electronic coherences can be found. The vertical grey pattern indicates the range corresponding to the inhomogeneous broadening of the 450 cm^−1^ and 680 cm^−1^ coherences, and the horizontal grey pattern indicates the range of the 840 cm^−1^ and 1300 cm^−1^ coherences. All four dimer coherences will be found in the overlapping square pattern region. (**e**) Emission dipole strengths of the four dimer electronic coherences. The color labeling of the FS bands and coherences in this figure is used throughout.

The spin 1/2  of the hole of each pair is coupled to the *p*-type orbitals (l=1) localized on the Se atoms [38,39]. These spin orbit interactions split each of the S bands into two sub-bands of states, with angular momentum: L=1± 1/2 state and L=1/2,3/2. The total angular momentum, L± the spin 1/2 of the *s*-type orbital localized on the Cd atoms, leads to an eight-fold degeneracy of the L=3/2 state and a four-fold degeneracy of the L=1/2 state. These states further undergo a loss of degeneracy due to crystal field and Coulomb interactions [36,38,39,40]. The $S_{3/2}$ state then forms a band of eight fine-structure (FS), singly excited electronic states, of which five are dark and three are bright, and the $S_{1/2}$ state forms a band of four FS states, of which all are bright. In this way, 4 bands of 24 FS states are formed per QD with energetic ordering: ${1S}_{3/2}$, ${1S}_{1/2}$, ${2S}_{3/2}$, and ${2S}_{1/2}.$ Figure 3d shows the stick spectrum of an ensemble-averaged monomer calculated over a 3 nm/8% ensemble of 4000 QDs [8,13].

When 2 QDs drawn from the size-dispersed ensemble of monomers are covalently bonded to form quasi-homodimers, the 24 FS states of each QD are coupled by interdot electronic Coulomb interactions to create a manifold of 48 FS states per dimer. Since the two QDs in a given dimer slightly differ in size, quasi-homodimers are formed: the quasi-isoenergetic bands of each QD are split by Coulomb interdot interactions into a higher and a lower dimer band. This creates eight bands of FS singly excited states per dimer. These bands are energetically ordered as: $1S_{3/2}^{L},$ $1S_{3/2}^{H}$, $1S_{1/2}^{L},$ $1S_{1/2}^{H}$, $2S_{3/2}^{L},$ $2S_{3/2}^{H},$ $2S_{1/2}^{L}$, and $2S_{1/2}^{H}$, although in dimers made from ensembles with larger $\bar{D}$ and/or size dispersion, the FS states of these bands interdigitate and the bands overlap [13,15]. 

The size differences of the two QDs assembled in each quasi-homodimer mean that they do not obey the optical selection rules of exact homodimers and that all the states will share the oscillator strength. Consequently, all singly excited dimer FS states will be bright, although some FS states will be almost dark. Figure 3b shows the stick spectrum calculated for the 3 nm/8% ensemble averaged over an ensemble of 4000 dimers [8,13]. 

Figure 3a shows the measured absorption spectra of the dimer solution along with the calculated absorption spectrum for the 3 nm/8% ensemble-averaged dimer and monomer, where the inhomogeneous broadening due to the finite size dispersion is taken into account. This figure also shows the spectral profile of the laser pulse used for the 2DES measurements. Only the $1S_{3/2}^{L}$ and the $1S_{3/2}^{H}$ dimer bands and the ${1S}_{3/2}$ monomer band fall within the laser pulse energy bandwidth. Tables S1 and S2 in Supplementary Materials, Section S3 present the calculated transition energies, inhomogeneous broadening and corresponding dephasing times, and transition dipole moments from the ground to the $1S_{3/2}^{L}$ and $1S_{3/2}^{H}$ band FS states in the dimer, and from the ground to the ${1S}_{3/2}$ band FS states in the monomer, averaged over 4000 dimer/monomer ensembles.

Since the laser pulses are short, several electronic states of the $1S_{3/2}^{L}$ and the $1S_{3/2}^{H}$ dimer bands are simultaneously excited, which led to a superposition of several excited FS electronic states. The electronic dimer coherences discussed in the Results Section are coherences between the FS excited states, either within (‘intra’-band) or between (‘inter’-band) the $1S_{3/2}^{L}$ and $1S_{3/2}^{H}$ bands. 

We specifically focus on four electronic coherences of the dimer, two intra-band and two inter-band, labeled by the periods of their oscillations, which are governed by the energy difference of the two FS states involved. The first intra-band coherences between the 3rd FS state and the degenerate 4th/5th FS states, all in the $1S_{3/2}^{L}$ band, have a frequency of $\approx 680\;{cm}^{-1}$. The second intra-band coherences between the degenerate 1st/2nd FS states and the degenerate 4th/5th FS states, all within the $1S_{3/2}^{L}$ band, have a frequency of $\approx 840\;{cm}^{-1}$. The first inter-band coherences between the degenerate 4th/5th FS states in the $1S_{3/2}^{L}$ band and the 11th FS state in the $1S_{3/2}^{H}$ band fall at $\approx 450\;{cm}^{-1}$. The second inter-band coherences between the degenerate 1st/2nd FS states in the $1S_{3/2}^{L}$ and the 11th FS state in the $1S_{3/2}^{H}$ band have a frequency of $\approx 1300\;{cm}^{-1}$. 

All these coherences, both intra- and inter-band, are interdot in character, due to the delocalization of the wavefunctions of the 1S FS states over the whole dimer [7]. The horizontal double-headed arrows superimposed onto the dimer stick spectrum (Figure 3b) identify these four coherences, and their calculated frequencies, periods, inhomogeneous dephasing times, and emission dipole strengths are presented in Table S3 of Section S3 of the Supplementary Materials. Throughout this work, the $450\;{cm}^{-1}$ coherences are labeled in cyan, and the $680\;{cm}^{-1}$, $840\;{cm}^{-1}$, and $1300\;{cm}^{-1}$ coherences in purple, green, and orange, respectively.

In the experiments, the pulse excites electronic states in the ${1S}_{3/2}$ band of the monomers that are also present in the sample, thereby creating monomer electronic coherences. The higher-energy monomer bands do not fall within the laser pulse energy bandwidth. Since five of the eight FS states in the ${1S}_{3/2}$ band are dark, or practically dark (see Table S2 of the Supplementary Materials, Section S3), only one type of electronic coherence will be produced non-negligibly in the monomers. This is the coherence between the 1st/2nd and 3rd FS states, which has a frequency of $180\;{cm}^{-1}$. This coherence, identified in Figure 3c with a double-headed arrow, will be labeled in yellow throughout this paper. The calculated frequency, period, inhomogeneous dephasing time, and emission dipole strength of this coherence are shown in Table S3.

The size dispersion of the QD ensemble causes an inhomogeneous broadening of the energies of the transitions between electronic states in the dimer and in the monomer, the values of which are presented in Tables S1 and S2 in the Supplementary Materials. In the time domain, the size dispersion leads to the dephasing of the coherences, which have a finite lifetime. In the ($T_{1},\omega_{3}$) time–frequency domain, the size dispersion, therefore, leads to dephasing along $T_{1}$ and to an inhomogeneous broadening of emission bands along $\omega_{3}$. The inhomogeneous broadening of the bands corresponding to the four dimer coherences on which we focus is represented as shaded areas on the time–frequency ($T_{1},\omega_{3}$) map reported in Figure 3d. Traces along *T*_2_ for points localized in a given $\omega_{3}$ band on the ($T_{1},\omega_{3}$) map will, therefore, exhibit beating periods that are characteristic of coherences (*i,j*) involving an excited state *j,* that emits in the range of $\omega_{3}$ values specified by the inhomogeneously broadened transition frequency ($\omega_{j}-\omega_{GS})$. 

Figure 3e shows the emission dipole strengths of the same four dimer electronic coherences. As was the case in [7], the rather monotonic distribution of these values results from the 8% size dispersion of the ensemble, which breaks the exact homodimer limit of fully dark and bright states. The rather even distribution of dipole strengths of the four coherences means that they will appear in the $T_{2}$ traces of points on the time–frequency ($T_{1},\omega_{3}$) maps with commensurate strength. This is a useful feature of disordered QD quasi-homodimers for applications in quantum technologies because it means that more coherences are available for implementing information processing. 

In this work, the partial polarizations in specific PMDs are modeled using a phase-modulated approach [41], which is numerically more straightforward to implement than a full simulation of the BOXCARS setup. In the experimental BOXCARS setup, phase modulation is not needed since the different PMDs are spatially separated by using a non-collinear setup [5,16,17,18]. On the other hand, a phase modulation of the train of pulses is experimentally implemented in 2DES collinear setups that measure action observables such as fluorescence or photocurrent [21,22,23,24,26,27,42]. 

To simulate the polarization response as measured in the BOXCARS setup, we compute the polarization of the ensemble of QD dimers subject to sequences of three collinear fs phase-modulated laser pulses. The electric field time profile of the pulse sequence is given as:

(2) $$ℇ\left(t\right)=\sum_{n=1}^{3}{ℇ}_{n}(t)$$

where ${ℇ}_{n}(t)$ is the electric field of each pulse, defined as:

(3) $${ℇ}_{n}\left(t\right)={ℇ}_{0}e^{\left(-\frac{{\left(t-t_{n}\right)}^{2}}{2\sigma_{n}^{2}}\right)}cos(\omega_{n}t+\phi_{n})$$

In Equation (3), ${ℇ}_{0}$ is the electric field strength, $\sigma_{n}$ is the width of the Gaussian envelope, $\omega_{n}$ is the carrier frequency, and $t_{n}$ is the time at which the nth pulse is centered, as shown in Figure 2a. In all the calculations, we use: ${ℇ}_{0}=8.775\times 10^{7}\;$ W/cm^2^, $\omega_{n}=$ 2.36 eV, and $\sigma_{n}=3.9\;fs$, for n=1, 2, 3, in agreement with the experimental values.

We modulate the carrier envelope phase of the pulse, $\phi_{n}$, for each set of delay times (*T*_1_, *T*_2_, *T*_3_). The modulation of $\phi_{n}$ is expressed as $\phi_{n}\equiv 2\pi k_{n}u$, where $k_{n}\equiv m_{n}/L$. By choosing the constants $m_{n}$ of each pulse as integer divisors of L, with $m_{1}\neq m_{2}\neq m_{3}$, after L repetitions for u varying from 1 to L, each of the carrier envelope phases, $\phi_{n}$, will have gone through a different number of complete cycles. 

The computations are repeated for ranges of delay times ($T_{1}$, $T_{2}$, $T_{3}$), modulating the carrier envelope phases of the pulses for each set of values ($T_{1}$, $T_{2}$, $T_{3}$). In this way, the polarization of the ensemble is computed as a function of the delay times and phase modulation, u, $P_{u}\left(T_{1},T_{2},T_{3}\right)$. The wave vector of each PMD is presented as a linear combination of the $m_{n}:$ $k_{l}=l_{1}m_{1}+l_{2}m_{2}+l_{3}m_{3}$, where the additional factors of u/L modulate the carrier envelope phase of the pulses. For the rephasing direction: $\left(l_{1},l_{2},l_{3}\right)=\pm \left(-1,+1,+1\right)$.

The polarization of the ensemble in a particular PMD is extracted by Fourier transforming along the phase modulations, u, and identifying the signal by the value of $k_{l}$.

(4) $$P_{k_{l}}\left(T_{1},T_{2},T_{3}\right)=\sum_{u=1}^{L}P_{u}\left(T_{1},T_{2},T_{3}\right)\cdot e^{-\frac{i2\pi }{L}k_{l}u}$$

One of these $P_{k_{l}}\left(T_{1},T_{2},T_{3}\right)$ values is shown in Figure 2b. $P_{u}\left(T_{1},T_{2},T_{3}\right)$ is calculated from the time-dependent ensemble density matrix, $\rho_{ens}$, as described in [8,13]:

(5) $$P_{u}\left(t\right)=Tr[\mu \;\rho_{ens}(t)]$$

where μ is the ensemble dipole matrix. The density matrix, $\rho_{ens}(t),$ is propagated along time, for each set of delay time and phase modulation parameters, using the ensemble Liouville approach [8,13].

The Liouville equation for the ensemble is given by [8]:

(6) $$i\hbar \frac{\partial \;}{\partial t}\rho_{ens}^{nm}\left(t\right)=\sum_{ij}L_{nm,ij}^{ens}\cdot \rho_{ens}^{ij}(t)$$

where $L^{ens}$, the ensemble Liouville matrix, is constructed by averaging the Hamiltonian matrices of the individual size-dispersed dimers over the ensemble and taking into account the size dispersion of the QDs. The ensemble Hamiltonian explicitly includes the interaction of the sequence of three laser pulses in the dipole approximation. Equation (6) is solved numerically using the Cash–Karp Runge–Kutta method. 

For a given set of delay times, (*T*_1_, *T*_2_, *T*_3_), the polarization is calculated for given sets, u, of carrier envelope phases, ($\phi_{1},\phi_{2},\phi_{3})$, using Equation (5), and parametrized in terms of these delay times using Equation (1).

(7) $$P_{u}\left(T_{1},T_{2},T_{3}\right)=P_{u}\left(t\right)$$

The polarization in each PMD is obtained by Fourier transform over *u* using Equation (4). Here, we focus on the rephasing of PMD. The computed data, $P_{reph}\left(T_{1},T_{2},T_{3}\right)$, are the time domain “cube” (Figure 2b) and are converted into the time–frequency domain by Fourier transforming the data along $T_{3}↔\omega_{3}$ (Figure 2c):

(8) $$P_{reph}(T_{1},T_{2},\omega_{3})↔\int e^{-i\omega_{3}T_{3}}P_{reph}(T_{1},T_{2},T_{3})dT_{3}$$

This produces a cube of data for the polarization of the ensemble in the rephasing direction in the time–frequency domain, as shown in Figure 2d. These computed data can be directly compared to the data measured in the BOXCARS setup in the time–frequency domain.

A rotating frame (RF) [43] is applied to the measured and calculated $P_{reph}(T_{1},T_{2},\omega_{3})$ (Equation (8)) [19]. A reference frequency, $\omega_{ref}$, is subtracted along $T_{1}$, thereby detuning the optical frequency.

(9) $$P_{reph}^{RF}=P_{reph}e^{i\omega_{ref}T_{1}}$$

In the computations, we take $\omega_{ref}\equiv \omega_{n}$, the carrier frequency of the pulse (Equation (3)). Working in the RF makes the electronic coherence between excited FS states clearer by removing the fast-beating coherences between the excited FS states and the GS, which have a much shorter dephasing time. This also allows for a less dense sampling in time along *T*_1_ and *T*_3_.

## 3. Results and Discussion

### 3.1. Comparison of Computed and Experimental Time–Frequency Polarization Maps

In our previous work [7], we showed that the electronic coherence response could be consistently characterized in BOXCARS polarization measurements of both solid-state and solution samples. Several electronic coherences between FS states in traces along *T*_2_ and their Fourier transforms (FTs) were characterized at specifically addressed points on measured and calculated rephasing frequency maps, $P_{reph}\left(\omega_{1},T_{2},\omega_{3}\right)$ (Equation (8)).

**Figure 4:**
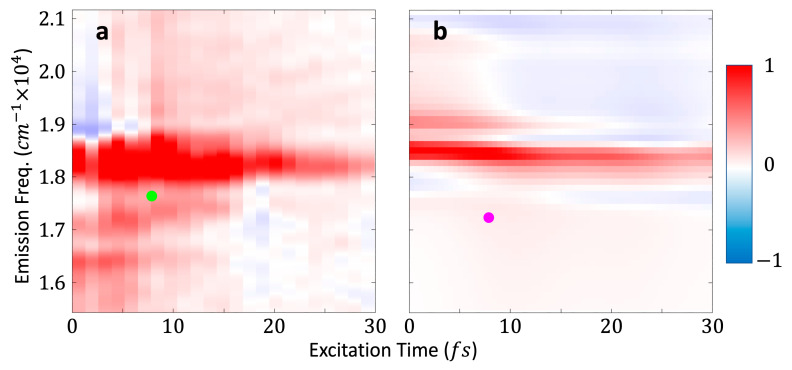
(**a**) Measured dimer sample and (**b**) calculated dimer ($T_{1}$,$\omega_{3}$) maps at $T_{2}$ = 20 fs after the rotating frame has been applied. The green and pink dots correspond to the locations where traces along *T*_2_ were taken, from which the FTs in Figure 5 were produced.

Figure 4 compares the real parts of the measured dimer sample (left) and the calculated dimer (right) rephasing ($T_{1},\omega_{3}$) time–frequency maps at *T*_2_ = 20 fs. In this range of *T*_1_ values, the main signal in both maps appears at an emission frequency around $\omega_{3}=$ 18,400 cm^−1^. This frequency corresponds to the transition energy between the ground state and the strongest dipole FS states in the first ${1S}_{3/2}^{L}$ dimer band. The experimental and calculated time–frequency maps are in very good agreement. The differences between the measured and calculated maps at emission frequencies below the main signal are attributable to Rayleigh scattering in the measurement.

*T*_2_ traces are extracted from the measured and computed maps at points corresponding to the green and pink dots in Figure 4 (see Supplementary Materials Section S4). Figure 4b shows a calculated dimer map, but the same trace was also taken through the calculated monomer maps.

These three traces, i.e., the measured averaged trace and the calculated dimer and monomer traces, are then Fourier-transformed:

(10) $$\int e^{-i\omega_{2}T_{2}}P_{reph}(T_{1},T_{2},\omega_{3})dT_{2}↔P_{reph}(T_{1},\omega_{2},\omega_{3})$$

**Figure 5:**
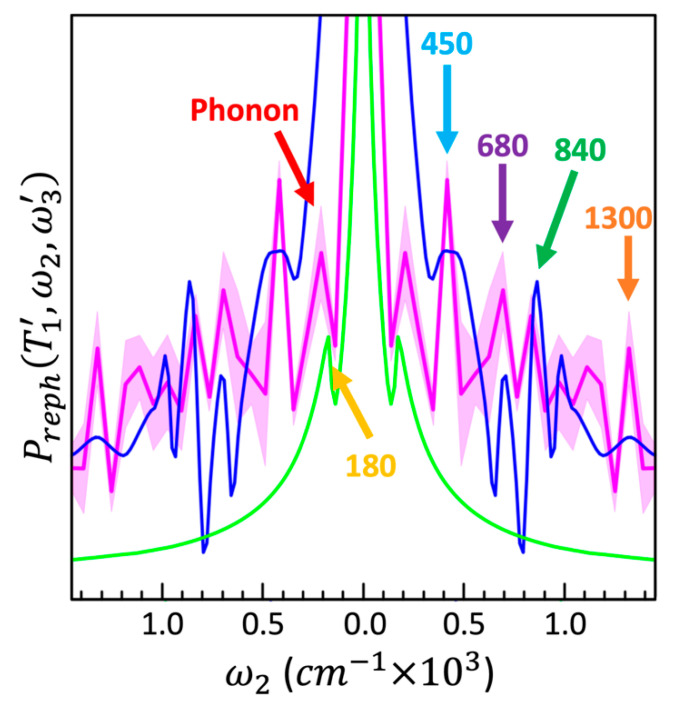
FTs of the time traces through the measured (Equation (10)) and calculated maps. The blue and green lines show the calculated dimer and monomer FTs, respectively, transformed along $T_{2}$ at the coordinate ($T_{1}=7.8\;fs$, $\omega_{3}=17,186\;{\mathrm{cm}}^{-1}$) on the time–frequency maps. The pink line is the mean of FTs from three measurements of the dimer solution sample, and the shaded area is their standard deviation, see Supplementary Materials Section S4 for more details.

Three replicates of the 2DES measurements were available. Therefore, we generated three measured FTs (one per replicate), from which a mean and standard deviation are produced. This mean measured FT is plotted with its standard deviation in Figure 5, along with the calculated dimer and monomer FTs.

The mean measured FT shown in Figure 5 has peaks corresponding to all the dimer coherences discussed in the theoretical model section. Their presence is made explicit by the comparison to the calculated dimer FT. The peaks in the mean measured FT at $\omega_{2}$ = 450 cm^−1^, 840 cm^−1^, and 1300 cm^−1^ are reliably characterized, with a narrow standard deviation. The peak at $\omega_{2}$ = 680 cm^−1^ has a larger standard deviation, however it is still distinctive. The region between $\omega_{2}\approx 900\;and\;1200\;{\mathrm{cm}}^{-1}$ has a significantly larger standard deviation and the peaks in this spectral range cannot be reasonably considered. The dimer coherence peaks in the mean measured FT align closely with their corresponding peaks in the calculated dimer FT. The alignment between the experimental and calculated FTs in the frequency domain is very good in the cases of the $\omega_{2}$ = 450 cm^−1^, 680 cm^−1^, and 1300 cm^−1^ peaks, and within the resolution of the $\omega_{2}\;$ points in the measured FT in the case of the 840 cm^−1^ peak.

The mean measured FT has a broad peak at $\omega_{2}\approx 200\;{\mathrm{cm}}^{-1}$ with a small standard deviation. This signal is primarily caused by the acoustic phonon beating [44]. This phonon signal is not present in the calculated dimer FT because our model does not include the coupling to the two phonon modes. However, in addition to the signal from the acoustic phonon, the 180 cm^−1^ monomer coherence also contributes to this broad peak, although this contribution is not resolved in the measured data. It should be noted that the coherence between the 1st/2nd and 3rd FS states in the $1S_{3/2}$ is also present in the dimer with a similar frequency. However, as the calculated dimer FT shows, this dimer coherence makes less of a contribution than the monomer coherence. 

Figure 5 shows that the four peaks in the measured mean FT at $\omega_{2}$ = 450 cm^−1^, 680 cm^−1^, 840 cm^−1^, and 1300 cm^−1^ are caused by coherences in the dimer, as there are no monomer coherences in their $\omega_{2}$ vicinity.

The dimer coherences discussed above are clearly defined in both the measured and calculated FTs in the time–frequency domain. Producing results in this way, as opposed to using ($\omega_{1},\omega_{3})$ frequency–frequency maps, leads to a drastic reduction in the number of the 2DES measurements needed. The $\omega_{3}$ coordinate where the measured traces are taken is consistent with the address in the frequency domain and depends upon the transition energies of the excited FS states involved in the coherences to the ground state. The coherences observed in Figure 5 can be best observed within a limited range of $T_{1}$ values, because at higher values of $T_{1}$, the fast-beating coherences between the ground state and the mono-excitons generated by the interaction with the first pulse is already de-phased. Hence, intra- and inter-band dimer coherences can be found in the time–frequency maps in a small range of short $T_{1}$ values and in a range of $\omega_{3}$ values which is defined by the transition energies from the FS states involved in the coherences to the GS.

The fact that the same electronic coherences can be characterized in the time–frequency domain as in the frequency–frequency domain of the 2DES experiments, and hence can be exploited in the directly measured data, is advantageous in two respects. The first is that the need for a post-processing step, such as Fourier transforming along $T_{1}↔\omega_{1}$, is removed. This yields a computational reduction of $O({N_{T}}_{1}log{N_{T}}_{1})$, where ${N_{T}}_{1}$ is the number of measurements along $T_{1}$. Following the removal of the FT step, the second advantage gained is the requirement for far fewer measurements along $T_{1}$ than are required to sufficiently resolve the FT needed to obtain frequency–frequency maps (see Supplementary Materials Section S5). As it has been discussed, measurements up to $T_{1}=10\;fs$ provide an adequate range in which the coherences can be exploited. This range is at least an order of magnitude smaller than the $T_{1}$ range required to resolve the FT of $T_{1}$ for frequency domain maps, according to the Nyquist limit. As discussed above, in the frequency maps, the ($\omega_{1},\omega_{3}$) address at which a coherence ‘*i-j*’ will beat along $T_{2}$ is given by the values of the ($\omega_{i}-\omega_{GS})$ and ($\omega_{j}-\omega_{GS})$ transition frequencies. In the time–frequency measurements discussed here, since only the third time interval, *T*_3_, is Fourier-transformed, only the addresses of the coherences along $\omega_{3}$ are resolved. All the intra-band and inter-band *i-j* coherences can be found in traces along $T_{2}$ in different ranges of $\omega_{3}\;$ but at the same value of $T_{1}$. This partial resolution of the addresses of the coherences significantly reduces the number of time delays that need to be sampled to characterize or exploit them for quantum technology applications. 

### 3.2. Modeling of Action-Based Photocurrent Response

The 2DES action-based fluorescence and photocurrent measurements [21,22,23,24,25,26,27,28,42] are a practical alternative to the polarization BOXCARS ones because they can be implemented with a much simpler collinear setup [5]. In addition, the photocurrent is a more appropriate choice of observable for quantum technology applications, as photocurrent measurements can be directly interfaced with classical electronics. Using the phase modulation approach described above, here, we report on the computed photocurrent response of electronic coherences of an ensemble of monomeric QDs. We set the pulse parameters to access the mono- and bi-exciton states of the QDs and show how electronic coherences involving bi-exciton states can be characterized in the $\left(T_{1},T_{2},\omega_{3}\right)$ photocurrent action signal in the double-quantum coherence (DQC) PMD. Note, while the DQC PMD spectra can be easily measured with a phase modulation setup [21], such measurements are harder with the BOXCARS setup because of the lack of fully reliable procedures to correctly phase the signal [45] and the possible contribution of a strong, spurious, non-resonant solvent response [46].

**Figure 6:**
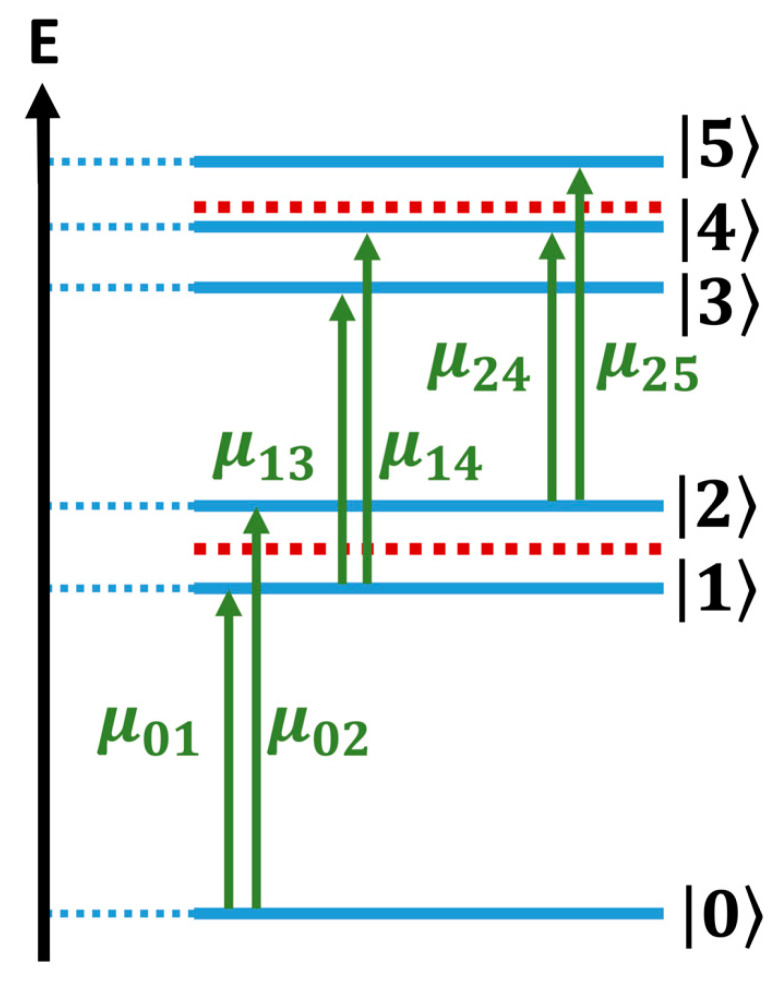
Energy level diagram for a monomeric QD excitonic system. The blue lines show the mono- and bi-exciton electronic states of the QD. The red dashed line shows the carrier frequency (and twice the carrier frequency) of the pulses (Equation (3)) used to compute the 2DES response.

Figure 6 shows the level structure of the mono- and bi-excitonic states of a single CdSe QD of 3 nm. It comprises the ground state $\left.\left|0\right.\right\rangle$, two mono-exciton states, $\left.\left|1\right.\right\rangle$ and $\left.\left|2\right.\right\rangle$, the S_1_ and the S_2_ states, and the three bi-exciton states, $\left.\left|3\right.\right\rangle$, $\left.\left|4\right.\right\rangle$, and $\left.\left|5\right.\right\rangle$, which correspond to a double-excitation to S_1_, to an excitation to S_1_ and to S_2_, and to a double-excitation to S_2_, respectively. These five excited states are represented by blue horizontal lines in the figure. The horizontal red dashed lines in Figure 6 show the carrier frequency (and twice the carrier frequency) of the laser pulse (Equation (3)) used in the simulations and the allowed dipole transitions are indicated with green vertical arrows. The calculated transition energies between these states averaged over an ensemble of 4000 monomeric QDs with 8% size dispersion, as well as the energies of the coherences between them, are presented in Table S4, along with the corresponding periods and dipole transition moments. Also presented are the dephasing lifetimes due to the size dispersion. 

The energy bandwidth of the laser pulses was selected so that the only transitions energetically allowed are those between the GS and the mono-exciton band and between the mono- and bi-exciton bands, as shown in Figure 6. Intra-band transitions between the states of the mono- or the bi-exciton bands are not resonant with the laser pulse.

Measurements of incoherent actions’ signals, such as fluorescence or, as we show here, photocurrent, require a setup with four pulses [22,23,26,47]. The nonlinear signals in specific PMDs are obtained using the phase modulation approach described above. 

**Figure 7:**
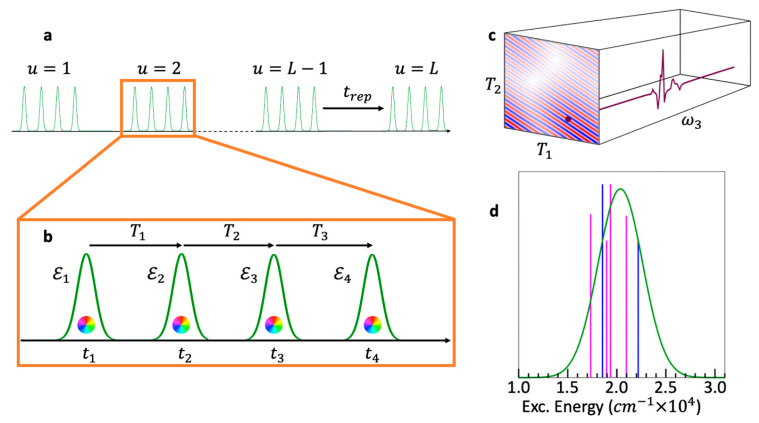
(**a**) Sequence of L phase modulation of 4 pulse trains used in the simulation. Each train has a different value of u, from 0 to L, which modulated the carrier envelope phase, and the trains are spaced by the laser repetition time, *t_rep_*. (**b**) The 4 pulses in each train are separated by the delay times, *T*_1_, *T*_2_, and *T*_3_, which are fixed in each sequence of L trains. The measurement is repeated using sequences with different sets of delay times. (**c**) The observable response in a specific PMD stored in a cube of data as a function of $T_{1},T_{2}\;,\;and\;\omega_{3}$. (**d**) The calculated stick spectrum with ground state–mono-exciton transitions in blue and mono-exciton–bi-exciton transitions in pink. The pulse envelope in the energy domain is superimposed onto the stick spectrum in green.

The action setup modeled is shown in Figure 7a,b [26]. A sequence of L trains of pulses is incident upon the ensemble, with each train being constituted of four pulses separated in time by the delay times $T_{1},T_{2}$, and $T_{3}$. Each train in the sequence has the same set of delay times, and the trains are spaced apart from one another by the repetition time, $t_{rep}$. The total electric field of each train is the same as in Equation (3), only the sum ran over n = 1, 2, 3, and 4. The action signal, from which the photocurrent is calculated, is recorded along $t_{rep}.$ For a given set of delay times ($T_{1},T_{2}$, $T_{3}),\;$ the carrier envelope phases of the four pulses are modulated from u=1 to L. The sequence is repeated using different values of the delay times, so the total photocurrent response is calculated as a function of the delay times and the phase modulation, $P_{u}\left(T_{1},T_{2},T_{3}\right)$. The total photocurrent is separated into the different PMDs using Equation (4), and these data are Fourier-transformed along $T_{3}$, $P_{PMD}\left(T_{1},T_{2},T_{3}\right)↔P_{PMD}(T_{1},T_{2},\omega_{3})$. This post-processing produces a cube of data, in which the photocurrent response in a specific PMD is stored as a function of $T_{1},T_{2},\omega_{3}$, as shown in Figure 7c. The analysis of this cube allows identifying and exploiting the coherences contributing to this PMD.

We take here for the pulse parameters: ${ℇ}_{0}=5\times 10^{-6}\;$ a.u. (8.775 × 10^5^ W/cm^2^), $\omega_{n}=$ 2.53 eV, and $\sigma_{n}=3.32\;fs,$ for n=1, 2, 3, and 4. The pulse envelope for these parameters in the energy domain is shown in Figure 7d, superimposed onto the stick spectrum of the ground state–mono-exciton and mono–bi-exciton transitions. In the simulations, L=170, $m_{1}=0,\;m_{2}=2,m_{3}=5,\;$ and $m_{4}=34$.

The observable response being computed is the photocurrent. We took the relaxation times in ranges typical for CdSe QDs [23,47]. The relaxation of the bi-exciton states to the mono-exciton states is in the sub-picosecond range, and the mono-exciton states are assumed to relax to the ground state with a lifetime of a dozen picoseconds. Consequently, the relaxation time from the $\left.\left|3\right.\right\rangle$ and $\left.\left|5\right.\right\rangle$ bi-exciton states to the $\left.\left|1\right.\right\rangle$ and $\left.\left|2\right.\right\rangle$ mono-exciton state is fixed to be 318 fs, with corresponding rates of: $\Gamma_{3\to 1}$ = $\Gamma_{5\to 2}$ = $1.3\times 10^{-2}\;eV$. The relaxation time from the $\left.\left|4\right.\right\rangle$ bi-exciton state to the $\left.\left|1\right.\right\rangle$ and $\left.\left|2\right.\right\rangle$ mono-exciton states is taken to be a little longer, at 636 fs, with a corresponding width in energy of: $\Gamma_{4\to 1}$ = $\Gamma_{4\to 2}$ = $6.5\times 10^{-3}\;\mathrm{eV}$. The relaxation time from the $\left.\left|1\right.\right\rangle$ and $\left.\left|2\right.\right\rangle$ mono-exciton states to the $\left.\left|0\right.\right\rangle$ ground state is taken to be 15.9 ps, with a corresponding width of: $\Gamma_{1\to 0}$ = $\Gamma_{2\to 0}$ = $2.6\times 10^{-4}\;\mathrm{eV}$. 

The total photocurrent response is calculated from the density matrix of the ensemble using the approximated Liouville matrix given in Equation (6), to which we added the relaxation rates defined above. Additionally, a decay term was added to the Liouville matrix to account for the dephasing of the coherences caused by phonon coupling with strength γ=0.005 eV. Both the relaxation of the bi-exciton to the mono-exciton states and of the mono-exciton states to the ground state contribute to the photocurrent signal [23,47]. The action signal response from the relaxation of state $\left.\left|m\right.\right\rangle$ to state $\left.\left|n\right.\right\rangle$ is computed as [47]:

(11) $${Resp}_{mn}=\int_{t_{4}}^{t_{aquisition}}\;dt\Gamma_{mn}Tr\left[\left.\left|n\right.\right\rangle \left\langle \left.m\right|\right.\rho \left(t\right)\left.\left|m\right.\right\rangle \left\langle \left.n\right|\right.\right]$$

The integral of Equation (11) is evaluated along the acquisition time of the experiment, which runs from the time of the fourth pulse in the sequence over the repetition time to the next sequence, $t_{rep}$, as shown in Figure 7a. In the simulations, $t_{rep}$ is approximated as ∞ since it is much longer than all the relaxation processes of the mono- and bi-exciton states to the ground state.

The total action signal response is:

(12) $$Resp=\int_{t_{4}}^{t_{aquisition}}\;dt\sum_{m,n}\Gamma_{mn}Tr\left[\left.\left|n\right.\right\rangle \left\langle \left.m\right|\right.\rho \left(t\right)\left.\left|m\right.\right\rangle \left\langle \left.n\right|\right.\right]$$

The total photocurrent calculated from Equation (12) is separated into its non-linear phase-matching components by Fourier transforming along the phase modulations (Equation (4)). We report here on the DQC PMD: $DQC=m_{4}+m_{3}-m_{2}-m_{1}$. As explained before, this is a phase-matching direction, challenging to be reliably measured in the BOXCARS setup, yet very appealing to quantify shifts of the energy correlation between two mono-excitons, in particular to study many-body effects and excited-state landscapes in a wide range of systems, including biomolecules and inorganic materials [19,21,48,49,50]. 

The calculated cube of data is then converted into the time–frequency domain by Fourier transforming along $P_{DQC}\left(T_{1},T_{2},T_{3}\right)↔P_{DQC}(T_{1},T_{2},\omega_{3})$.

**Figure 8:**
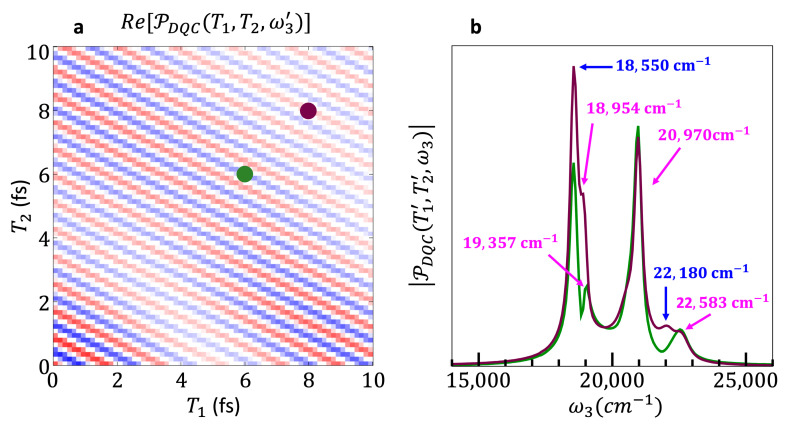
(**a**) ($T_{1},T_{2}$) Time map of the real part of the photocurrent response in the DQC PMD for a value of $\omega_{3}=20,000\;{\mathrm{cm}}^{-1}$. (**b**) Traces along $\omega_{3}$ at the points indicated in (**a**) with a green dot, ($T_{1},T_{2}$) = (6.0 fs,6.0 fs), and a burgundy dot, ($T_{1},T_{2}$) = (8.0 fs,8.0 fs). The signal corresponding to ground–mono-exciton state coherences is identified with blue arrows, and the signal corresponding to mono–bi-exciton state coherences is identified with pink arrows.

Figure 8a shows a ($T_{1},T_{2}$) time map of the real part of the photocurrent response in the DQC PMD for a value of $\omega_{3}=20,000\;{\mathrm{cm}}^{-1}$. The pathways contributing to the DQC PMD using a third-order perturbative approach are enumerated as double-sided Feynman diagrams by Damtie et al. [47]. We do not use a perturbative approach here, instead we are computing the photocurrent response from the dynamics of the density matrix (Equation (6)). However, the pulse strength used in the simulation is sufficiently weak that the third-order perturbative treatment is a good approximation of the exact time-dependent response and provides good insights into the excitation pathways contributing to the signal. The double-sided Feynman diagrams which contribute to the DQC PMD all have in common that the first pulse excites the ket from the ground state to a mono-exciton state, and the second pulse excites this ket from the mono-exciton state to a bi-exciton state. This means that for the duration of $T_{1}$, the system will be in a coherence between the ground state and a mono-exciton state, and that for the duration of $T_{2}$, the system will be in a coherence between the ground state and a bi-exciton state. These features clearly appear in the time map of Figure 8a. If one compares the number of oscillations along the $T_{1}$ axis to those along the $T_{2}$ axis, it is clear that there are more beatings in the same time along $T_{2}$ than $T_{1}.$ This reflects the fact that the ground–bi-exciton coherences have much shorter periods, about two times shorter, than the ground–mono-exciton coherences (see Table S4). 

Figure 8b shows the absolute values of the traces along $\omega_{3}$ of two points on the ($T_{1},T_{2}$) time map. The Feynman diagrams [47] show that the third pulse in the train can either relax the ket from the bi-exciton state to a mono-exciton state or excite the bra from the ground state to a mono-exciton state. This means that for the duration of $T_{3},\;$ the system can either be in a coherence between the ground and a mono-exciton state or between a mono-exciton and a bi-exciton state. This is shown in Figure 8b, in which the fast-beating responses corresponding to ground–mono-exciton as well as ground–bi-exciton coherences are identified. 

Note that the signal of the two traces plotted in Figure 8b, while being dominated by the same primary frequencies, differs in the specific coherences which can be identified. This small but important variability of the coherences in the $\omega_{3}$ traces of different ($T_{1},T_{2}$) points means that a comprehensive collection of coherence data still requires repetitions for a small number of $T_{1}$ and $T_{2}\;$ values, albeit far fewer than would be needed if Fourier transforms along these delay times were required. In addition to the inhomogeneous broadening resulting from the size dispersion of the ensemble, the peaks in Figure 8b are broaden by the coupling to the phonon modes. 

The analysis of the computed photocurrent response in an additional PMD, the DQC PMD, shows that coherences involving bi-exciton states can be observed in the action signal photocurrent response of QD ensembles to 2DES. As in the case of the polarization response, directly measured data in the $\left(T_{1},T_{2},\omega_{3}\right)$ domain are usable without the need for additional post-processing by Fourier transforms along $T_{1}$ and $T_{2}$. A sampling of the delay time, *T*_3_, for fixed values of *T*_1_ and *T*_2_ enables to characterize all the coherences found in the pathways contributing to the DQC PMD. This, in turn, means that fewer measurements along these delay times are needed. Furthermore, these results show that the $\left(T_{1},T_{2},\omega_{3}\right)$ is the appropriate domain for looking at coherences in observables in the DQC PMD.

## 4. Conclusions

We have shown that the electronic coherences resulting from the excitation of ensembles of size-dispersed QDs and QD dimers by sequences of fs broad-bandwidth laser pulses, as in 2DES, can be observed and characterized in the directly measured time–frequency domain. This is the case for both polarization and action-based (here, photocurrent) measurements. For the case of the cube of data that depends on three delay times, the electronic coherences are characterized by robust and distinct beating patterns in the traces of the signal, as a function of one delay time at a single point in the time–frequency domain of the other two delay times. In the case of polarization measurements, we obtained very good agreement between the modeled and experimental ($T_{1\;},\;\omega_{3}$) time–frequency maps in the rephasing PMD, and in the $T_{2}/\omega_{2}\;$ traces along these maps.

Compared to the conventional ($\omega_{1\;},\;\omega_{3}$) frequency maps, the advantage of directly exploiting time–frequency data leads to a considerable reduction of the number of time intervals that need to be sampled. This is because, in a time–frequency map, the addresses of the coherences are only partially resolved. All the coherences between excited states *i* and *j*, which have a common state *j*, beat in the traces of points with the same *T*_1_ value. By fixing $\omega_{3}$ to be within the inhomogeneous broadening of the transition *j* to the GS, and recording a trace at a point (*T*_1_, $\omega_{3})$ along *T*_2_, one can characterize, at the same time, all the beating frequencies of a family of coherences that involve the same emitting state *j* in the chosen PMD, because the absorbing states *i* are not resolved along *T*_1_.

That several coherences are simultaneously accessible in the directly measured time–frequency domain is a huge advantage for the exploitation of QD electronic coherences in quantum technologies for parallel information processing. The savings in fewer measurements and less computation time will greatly enhance the advantage of logical operations encoded onto coherences over classical logic operations. Action-based photocurrent measurements of electronic coherences are a step further in coherence exploitation since they pave the way for QD devices to be integrated into classical architectures.

## Data Availability

Not applicable.

## Supplementary Materials

The following supporting information can be downloaded at: https://www.mdpi.com/article/10.3390/nano13142096/s1, Section S1: Experimental details on the 2DES set-up. Section S2: Materials and method for the synthesis of CdSe QDs and dimers preparation. Section S3: Tables of the computed energy levels of the QD dimers. Section S4: Extraction of *T*_2_ traces and their corresponding *ω*_3_ FTs from the measured and calculated time-frequency maps. Section S5: Scaling of data processing for time-frequency responses (*T*_1_, *T*_2_, *ω*_3_) and frequency-frequency responses (*ω*_1_, *T*_2_, *ω*_3_). References [7,19,20,30,31] are cited in the supplementary materials.
