# Supplementary material for: Time–Frequency Signatures of Electronic Coherence of Colloidal CdSe Quantum Dot Dimer Assemblies Probed at Room Temperature by Two-Dimensional Electronic Spectroscopy
Source: Nanomaterials (Basel). 2023 Jul 18;13(14):2096. doi: 10.3390/nano13142096 (PMC10384478; doi:10.3390/nano13142096)
Supplement: Supplementary file 1 [file nanomaterials-13-02096-s001.zip › nanomaterials-2504766-supplementary.pdf]

Supporting Material

Time-frequency signatures of electronic coherence of colloidal CdSe quantum dot dimer assemblies probed at room temperature by 2-dimensional electronic spectroscopy

James R. Hamilton[1], Edoardo Amarotti[2,+], Carlo N. Dibenedetto[3,4], Marinella Striccoli[3] R.D. Levine[5], Elisabetta Collini[2], F. Remacle[1*]

1 Department of Theoretical Physical Chemistry, University of Liège, B4000 Liège, Belgium

2 Department of Chemical Sciences, University of Padova, Padova, Italy.

3 CNR-IPCF SS Bari, c/o Chemistry Department, University of Bari Aldo Moro, Bari, Italy

4 Chemistry Department, University of Bari Aldo Moro, Bari, Italy

5 The Fritz Haber Research Center for Molecular Dynamics and Institute of Chemistry, The Hebrew University of Jerusalem, Jerusalem 91904, Israel

+Present address: Division of Chemical Physics and NanoLund, Lund University, P.O. Box 124, Lund 22100, Sweden

*Corresponding author: Francoise Remacle, fremacle@uliege.be

Section S1: Experimental details on the 2DES set-up
Section S2: Materials and method for the synthesis of CdSe QDs and dimers preparation
Section S3: Tables of the computed energy levels of the QD dimers
Section S4: Extraction of T_2 traces and their corresponding ω_3 FTs from the measured and calculated time-frequency maps.
Section S5: Scaling of data processing for time-frequency responses (T_1,T_2, ω_3) and frequency-frequency responses (ω_1,T_2, ω_3).

Section S1: Experimental details on the 2DES set-up

2DES measurements were conducted with the setup described in Ref.[1] and the experimental data have been fully reported and analyzed in ref.[2].

Briefly, the output of an 800 nm, 3kHz Ti:Sapphire laser system (Coherent Libra) is converted into a broad visible pulse in a non-collinear optical amplifier (Light Conversion TOPAS White). The spectrum was centered at 18100 cm^{-1} (552 nm) and had a spectral width of about 1000 cm^{-1}. The transform-limited condition for the pulses at the sample position is achieved through a prism compressor coupled with a Fastlite Dazzler pulse shaper for the fine adjustment. The pulse duration, optimized through FROG measurements, is about 10 fs, as shown in Figure S1. The 2DES experiment relies on the passively phase stabilized setup, where a suitably designed 2D grating splits the incoming pulse into four identical phase-stable beams (three exciting beams and a fourth beam further attenuated of 3 orders of magnitude and used as Local Oscillator, LO) arranged at the four edges of a square. This phase-matching geometry is called BOXCARS.[3]

Time delays between pulses (T_1 and T_2) are modulated by pairs of wedges (4° CaF_2 wedges) with a final temporal resolution of 0.07 fs, while the third dimension is directly acquired in the frequency domain, ω_3, using a CCD camera. The following experimental parameters were used: (i) the exciting energy on the samples was set to about 7 nJ per pulse; (ii) the beam waist was about 100 µm; (iii) the population time (T_2) was scanned from 0 to 500 fs, in steps of 5 fs; (iv) all the measurements have been performed at ambient temperature; (v) each experiment was repeated at least five times to ensure reproducibility.

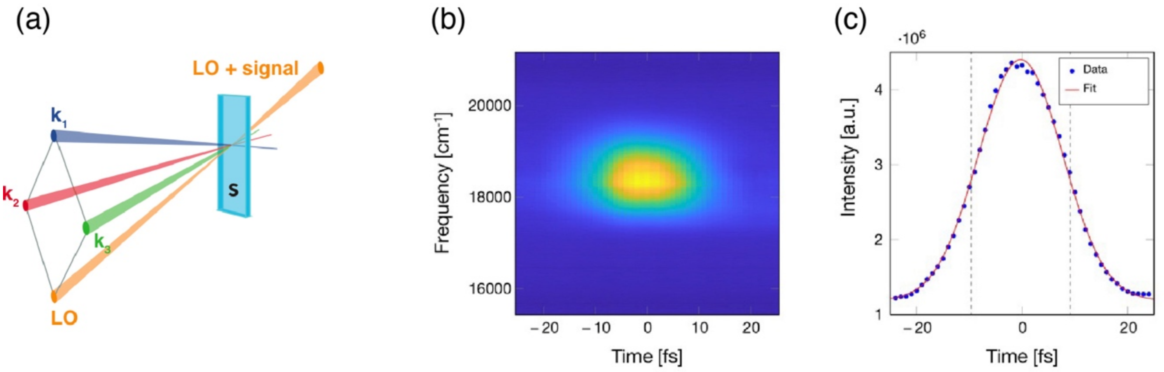

Figure S1. (a) Schematic representation of the BOXCARS phase-matching geometry. (b, c) Characterization of the pulse used in the experiments: (b) frequency resolved optical gating (FROG) measurement and (c) the associated signal integrated along the frequency axis. Blue dots are experimental points, and the red line is the Gaussian fit. The full-width half maximum (FWHM) of the pulse is quantified of about 10 fs.

S2 Materials and method for the synthesis of CdSe QDs and dimers preparation

Materials. Cadmium oxide (CdO, 99.5%), selenium (Se, 99.99%), oleic acid (OLEA, 90%), trioctylphosphinoxide (TOPO, 99%), tributylphosphine (TBP, 99%), hexadecylamine (HDA, 90%), butylamine (BUA, 99%), 1,3 propanedithiol (pDT, 99%), 1,16 hexadecanedithiol (hdDT, 99%). All chemicals were used as received, without any further purification or distillation. Ethanol (≥ 99,8%), and hexane (anhydrous ≥ 99%) were used at analytical grade, unless otherwise specified, and purchased from Sigma-Aldrich.

Synthesis of CdSe QDs. A mixture of 0.127 g of CdO (1 mmol) and 1 mL of OLEA was heated at 90°C in a three necked flask under vacuum to eliminate moisture and then heated up to 260°C under nitrogen to completely decompose the CdO red powder into the Cd-oleate complex (pale yellow solution). The Cd-oleate mixture was then cooled down to 85°C and kept under nitrogen. TOPO (9 g, 23 mmol) and HDA (9 g, 37 mmol) were put at 110°C under vacuum for 1 hour, cooled down to 85°C and transferred into the Cd oleate flask. The temperature was then increased up to 300°C and 2 mL of TBP were injected at 280°C followed by the Se precursor solution (0.394 g of Se,5 mmol, dissolved in 4.5 mL of TBP) injection at 295°C (injection temperature). Soon, after the injection, the temperature was lowered down to 270°C (growth temperature) and the flask was kept at this temperature under stirring for 30 seconds (reaction time), for the synthesis of the QD sample. To quench the QD growth the reaction mixture was cooled down. A purification procedure was carried out by addition of ethanol both to precipitate and wash the QDs and to remove unreacted products. The final sample was redispersed in 4 mL of hexane, resulting in concentration of 10^{-4}M.

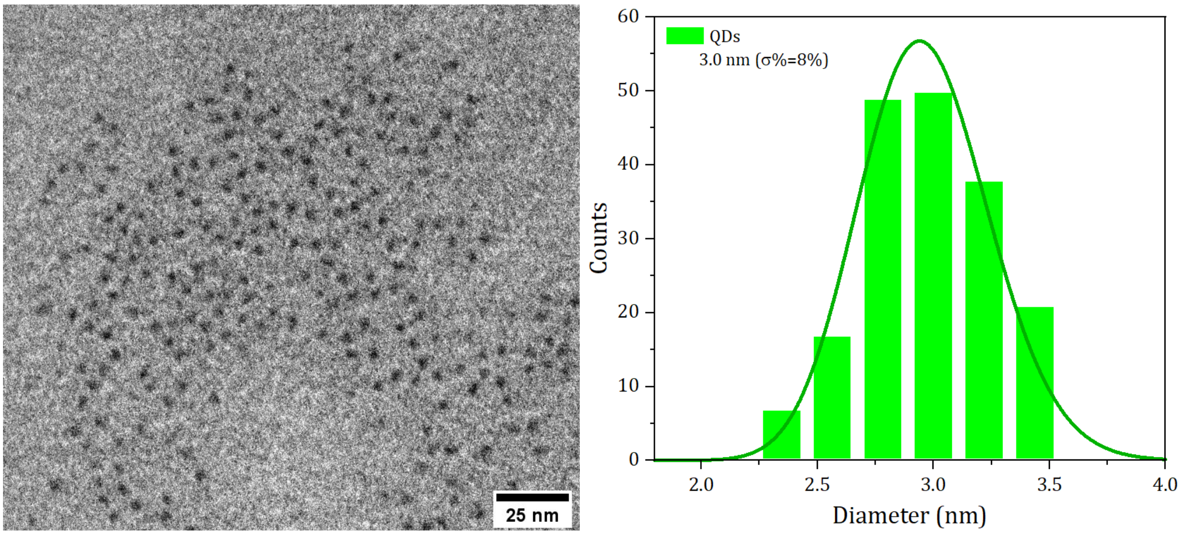

Figure S2: TEM micrograph of the QD sample synthetized and its relative size statistical analysis.

Statistical analysis of the size (QD average size (diameter) and size distribution) of the samples was performed by using of a freeware image analysis program, ImageJ. The percentage relative standard deviation (σ%) was calculated for each sample, providing information on the QD size distribution. Its value is based on the distribution of size compared to the average value and is expressed as a percentage and results in CdSe QDs with mean diameter $\bar{D}$ = 3 nm and a narrow size distribution σ = 8%, making any further purification procedure not necessary.[4]

QD dimers fabrication by two steps functionalization with butylamine and alkyl dithiols.
QD colloidal solution was diluted in hexane at $5\cdot10^{-7}$M was and stock solutions of pDT at $3.3\cdot10^{-3}$M was prepared.
Dimers preparation by a two steps approach were carried out for the QD sample, see ref [5]. First, QDs ($5\cdot10^{-7}$M) were treated with a solution of BUA ($2.5\cdot10^{-2}$M) at BUA:QD molar ratio 500:1. Addiction of pDT:QD molar ratio of 50:1 allows the sef-assembly of QDs in molecular assemblies up to 8 QDs linked together, i.e. dimers.
The crystallographic analysis of the Fast Fourier Transforms (FFTs) of the HR-TEM images indicates a wurtzite structure (ICSD 415785) of the nanocrystals constituting the assembly,

while the nearest surfaces involved in the linkage between the QDs are the (101) faces, as reported in ref. 28 of the main text.

Samples were diluted to 1:60 in hexane for TEM grid preparation and statistical analysis of dimer yield (DY).

The DY in each sample has been calculated as the ratio between the number of dimers counted in each TEM micrograph (at least 15 different images were considered for each sample) over the total number of particles. In our analysis, assemblies formed of less than 6-8 QDs, for which it is still possible consider the molecular nature of the assembly, are taken into account. In this sense, the obtained DY overcomes the value of 60%. The remaining fractions are given by either single QDs or larger aggregates. It is worth to note that the aggregates composed of tens or hundreds of nanoparticles were not considered in the calculation of the DY, due to the large error in the determination of the correct number of QDs, then underestimating the actual value of the DY.

Section S3 : Tables of the computed electronic levels for QD dimers and monomers

Table S1: Calculated FS state transition energies, inhomogeneous broadenings and dephasing times, and dipole moments for the ground state to the $1S^L_{3/2}$ *and* $1S^H_{3/2}$ *bands of FS states averaged over a dimer ensemble of 4000 3nm/8% dimers.*

i	Band	j	Transition Energy (cm^{-1})	Inhomogeneous Broadening (cm^{-1})	Inhomogeneous Dephasing Time (fs)	Transition dipole Moment (a.u.)
0	$1S^L_{3/2}$	1	17660	390	85	7.0
0		2	17660	390	85	7.0
0		3	17820	393	85	7.1
0		4	18500	433	77	12.1
0		5	18500	433	77	12.1
0		6	18630	436	76	10.6
0		7	18690	458	73	1.3
0		8	18710	453	74	2.4
0	$1S^H_{3/2}$	9	18880	473	70	5.2
0		10	18880	472	71	5.5
0		11	18950	481	69	7.1
0		12	19690	521	64	1.1
0		13	19700	528	63	0.8
0		14	19760	516	65	1.9
0		15	19800	538	62	1.1
0		16	19480	551	61	0.6

Table S2: Calculated FS state transition energies, inhomogeneous broadenings and dephasing times, and dipole moments for the ground state to the $1S_{3/2}$ and $1S_{1/2}$ bands of FS states averaged over a monomer ensemble of 4000 3nm/8% QDs.

i	Band	j	Transition Energy (cm^{-1})	Inhomogeneous Broadening (cm^{-1})	Inhomogeneous Dephasing Time (fs)	Transition dipole Moment (a.u.)
0	$1S_{3/2}$	1	18150	580	58	14.5
0		2	18150	580	58	14.4
0		3	18330	580	58	14.2
0		4	19200	-	-	0
0		5	19200	-	-	0
0		6	19300	684	49	0.8
0		7	19300	684	49	0.8
0		8	19330	-	-	0
0	$1S_{1/2}$	9	20700	670	50	6.3
0		10	20800	680	49	5.64
0		11	20800	680	49	5.64
0		12	21000	704	47	0.6

Table S3: Calculated transition energies (approximate) between dimer FS states with the corresponding coherence periods, inhomogeneous dephasing times and emission dipole strengths for the 5 types of coherences discussed in the result section of the main text.

Band	i	Band	j	Transition Energy (cm^{-1})	Period (fs)	Inhomogeneous Dephasing Time (fs)	Dipole Strength (a.u.)
Dimer							
$1S^L_{3/2}$	3	$1S^L_{3/2}$	4	680	49	266	85.8
			5	680	49	266	85.8
	1		4	840	40	266	84.9
			5	840	40	266	84.9
	2		4	840	40	266	83.9
			5	840	40	266	83.9
$1S^L_{3/2}$	1	$1S^H_{3/2}$	11	1300	26	285	50.2
	2			1300	26	285	49.7
	4		11	450	74	251	86
	5			450	74	251	86
Monomer							
$1S_{3/2}$	1	$1S_{3/2}$	3	180	185	10000	205
	2			180	185	10000	205

Table S4: Transition energies between ground, mono- and biexciton states in the system. Also given are the corresponding periods, inhomogeneous dephasing times and dipole strengths.

i	j	Transition Energy (cm^{-1})	Period (fs)	Inhomogeneous Dephasing Time (fs)	Transition Dipole Moment (a.u.)
0	1	18550	1.80	89	14.4
0	2	22180	1.50	42	10.2
0	3	37908	0.88	45	0.0
0	4	39521	0.84	29	0.0
0	5	41134	0.81	21	0.0
1	3	19357	1.72	89	14.4
1	4	20970	1.59	42	12.0
1	5	22583	1.48	27	0.0
2	3	15727	2.12	71	0.0
2	4	17340	1.92	89	12.2
2	5	18954	1.76	42	10.2

Section S4: Extraction of T_2 traces and their corresponding ω_3 FTs from the measured and calculated time-frequency maps.

T_2 traces were taken through the measured maps for values of ω_3 =17500 to 18100 cm^{-1}, for the same T_1 = 7.8 fs. This range of measured traces are then averaged into a single trace with effective coordinates (T_1=7.8 fs, ω_3 =17800), indicated on Figure 4 (a) with a green dot. T_2 traces were taken through the calculated dimer and monomer maps at the coordinates (T_1=7.8 fs, ω_3 =17200 cm^{-1}), indicated on Figure 4 (b) with a pink dot.
Four data sets of the measurements were taken on the solution sample; however, one was discarded as an outlier. To quantify the signal to noise ratio in the beating frequencies of the electronic coherences along T_2, we took the Fourier Transform of the experimental traces described above for the three remaining replicates of the measurement of the same sample. Each individual trace $f_i(T_2)$ was Fourier transformed, and the mean- value $\bar{f}(\omega_2) = (1/3)\sum_{i=1}^{3} f_i\,(\omega_2)$ and the standard deviation are computed. They are plotted in Figure 5 along with the FTs of the calculated dimer and monomer traces.

Section S5: Scaling of data processing for time-frequency responses (T_1,T_2, ω_3) and frequency-frequency responses (ω_1,T_2, ω_3).

The Fast Fourier Transform (FFT) scales like O(N log N) where N is the number of points. Using a single Intel Core i7-4930K Processor, a FFT of N_{T1}=1200 values of T_1 takes approximately 2.1x10^{-5} seconds. This FFT has to be repeated for each combination of T_2 and ω_3. For N_{T2} =101 measurements of T_2 and $N_{\omega 3}$ =256 measurements of ω_3, this stage of the data processing takes approximately 0.5 seconds for one phase matching direction (PMD) and scales as O(N_{T2}*$N_{\omega 3}$*N_{T1}*logN_{T1}).

Supplementary References

1. Bolzonello, Luca, Andrea Volpato, Elena Meneghin, and Elisabetta Collini. "Versatile Setup for High-Quality Rephasing, Non-Rephasing, and Double Quantum 2d Electronic Spectroscopy." *Journal of the Optical Society of America B* 34, no. 6 (2017): 1223-33.
2. Hamilton, James R., Edoardo Amarotti, Carlo Nazareno Dibenedetto, Marinella Striccoli, R. D. Levine, Elisabetta Collini, and F. Remacle. "Harvesting a Wide Spectral Range of Electronic Coherences with Disordered Quasi-Homo Dimeric Assemblies at Room Temperature." *Advanced Quantum Technologies* 5 (2022): 2200060.
3. Eckbreth, Alan C. "Boxcars: Crossed-Beam Phase-Matched Cars Generation in Gases." *Applied Physics Letters* 32, no. 7 (2008): 421-23.
4. Roberge, Adam, John H. Dunlap, Fiaz Ahmed, and Andrew B. Greytak. "Size-Dependent Pbs Quantum Dot Surface Chemistry Investigated Via Gel Permeation Chromatography." *Chemistry of Materials* 32, no. 15 (2020): 6588-94.
5. Dibenedetto, Carlo Nazareno, Elisabetta Fanizza, Rosaria Brescia, Yuval Kolodny, Sergei Remennik, Annamaria Panniello, Nicoletta Depalo, Shira Yochelis, Roberto Comparelli, Angela Agostiano, Maria Lucia Curri, Yossi Paltiel, and Marinella Striccoli. "Coupling Effects in Qd Dimers at Sub-Nanometer Interparticle Distance." *Nano Research* 13, no. 4 (2020): 1071-80.
